# Anatomical Variations in Mandibular Molars: Focus on Midmesial Canals

**DOI:** 10.7759/cureus.61711

**Published:** 2024-06-05

**Authors:** Khyati Manik, Anuja Ikhar, Aditya Patel, Manoj Chandak, Jay Bhopatkar, Pratik Rathod, Priyanka R Bhojwani

**Affiliations:** 1 Department of Conservative Dentistry and Endodontics, Sharad Pawar Dental College and Hospital, Datta Meghe Institute of Higher Education and Research, Wardha, IND

**Keywords:** midmesial canal, endodontics, mandibular molar, obturation, magnification

## Abstract

Endodontic therapy focuses on the root canal system to treat infected or damaged pulp tissue within the tooth, ultimately preserving the tooth and restoring its function. The root canal space should be cleaned with the use of proper instruments and chemical disinfectants to eradicate infected pulpal tissue and its remnants. The failure of endodontic therapy is attributed to a lack of understanding of the differences in anatomy among teeth, as evidenced by research. Canals are identified, and endodontic treatment is facilitated by the use of dental operating microscopes. Therefore, to achieve a favorable endodontic result, it is imperative to use all available methods to identify additional aberrant root canals. Failure to detect and adequately treat the midmesial canal (MMC) can lead to persistent infection, treatment failure, and the need for retreatment. This case underscores the importance of meticulous assessment and advanced techniques in treating complex canal configurations, ultimately leading to favorable outcomes in endodontic therapy. The MMC, a challenging anatomical feature, was located through careful clinical and radiographic examination. Advanced techniques, including ultrasonic activation and meticulous instrumentation, were employed to navigate and clean the canal effectively. Sodium hypochlorite irrigation and passive ultrasonic activation were utilized for thorough disinfection. The MMC was sealed with biocompatible materials, ensuring comprehensive obturation of the root canal system.

## Introduction

The primary objective of root canal therapy is the thorough mechanical and chemical cleansing of the entire pulp space from microorganisms, dentin debris, and remnant pulp tissue [1]. Various reasons for endodontic treatment failure have been found to include missing canals, incomplete instrumentation, insufficient biomechanical preparation and cleaning of the root canals, and subsequent poor filling of the root canals [2]. A fundamental aspect of endodontic therapy is achieving proper access to the pulp chamber, which not only grants entry to the root canal orifices but also provides an ideal viewpoint for observing the chamber floor. This step is crucial as it facilitates the identification of any variations in the number and position of root canals [3].

Anatomical variations in the mandibular first molar are well-documented and have significant implications for endodontic treatment outcomes. These variations primarily involve the root canal system and can present challenges during root canal therapy. One common anatomical variation is the presence of additional canals, such as the midmesial canal (MMC). This canal, located between the mesiobuccal and mesiolingual canals, has been increasingly recognized with the advent of advanced imaging techniques like CBCT. Its presence can complicate treatment if not properly identified and treated. Another common variation is the presence of extra roots, such as the radix entomolaris and radix paramolaris, which can complicate instrumentation and increase the risk of untreated canal systems. Additionally, curved canals, accessory canals, and C-shaped configurations are frequent findings, demanding careful negotiation and thorough disinfection.

Pomeranz in the year 1981, distinguished between the independent, confluent, and finned middle mesial canals. If such canal anomalies are not detected, this can lead to endodontic failure [4]. Effective management of the MMC typically involves thorough cleaning, shaping, and disinfection of the MMC to remove any pulp tissue, debris, or bacteria that may be present. This often necessitates the use of specialized endodontic instruments, such as ultrasonic tips or nickel-titanium rotary files, capable of negotiating the narrow and tortuous canal pathway. Additionally, adjunctive irrigation with antimicrobial solutions and passive ultrasonic activation can enhance disinfection within the MMC and its associated ramifications.

Locating the MMC in mandibular first molars poses a challenge in routine endodontic practice due to various factors. Its position between the mesiobuccal and mesiolingual canals complicates direct visualization, especially considering the limited visibility within the pulp chamber. Moreover, the narrow and intricate nature of the canal system may hinder effective instrumentation, limiting the ability to negotiate and explore the MMC thoroughly. Overall, the difficulty in routinely locating the MMC underscores the need for advanced visualization aids, such as dental loupes and cone-beam computed tomography, as well as comprehensive training to enhance diagnostic accuracy and improve endodontic outcomes.

Nowadays, the magnification holds great significance in determining the exact identification of root canals and thus result of dental treatment [5]. "Dental microscopes, and surgical loupes" are some of them that help to accurately identify or locate the root canals. Dental loupes play a crucial role in aiding endodontists and general dentists in locating anatomical variations such as the MMC in mandibular first molars. In the case of the MMC, which can be challenging to detect due to its location between the mesiobuccal and mesiolingual canals, dental loupes provide improved clarity and depth perception, enhancing the likelihood of its identification. The magnified view afforded by loupes enables the clinician to thoroughly inspect the pulp chamber and root canal system, increasing the chances of detecting subtle deviations from the typical canal morphology .The successful endodontic treatment of a mandibular first molar with three canals in the mesial root is detailed in this report.

## Case presentation

**Figure 1:**
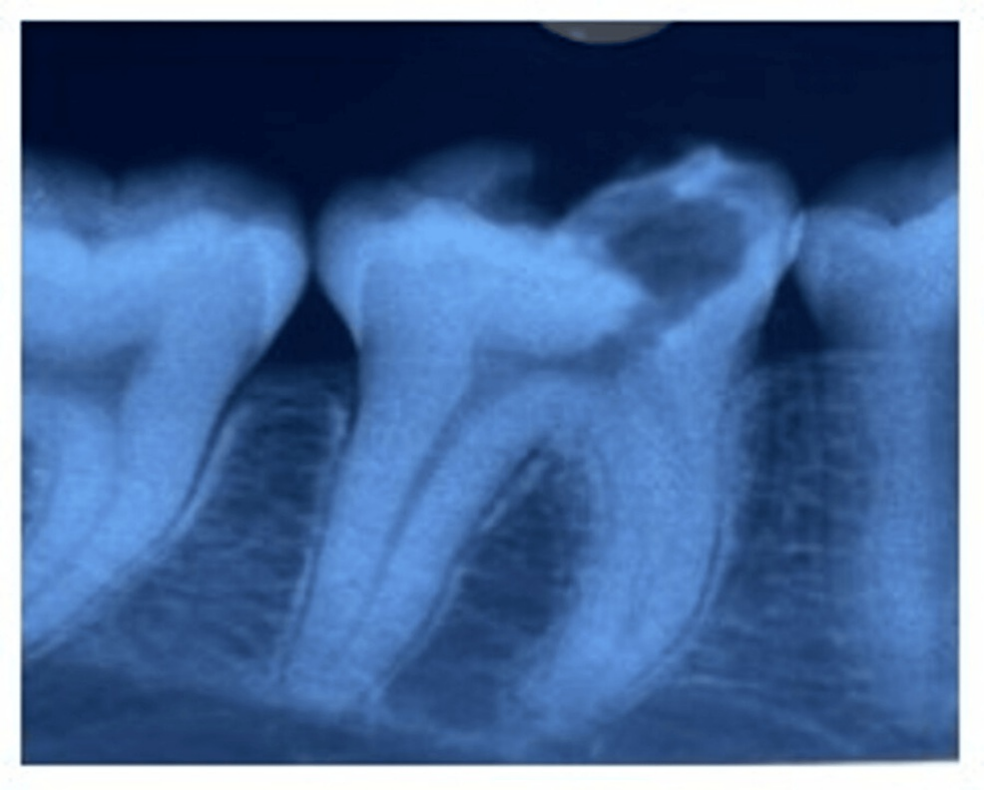
Preoperative radiograph Image by Dr. Khyati Manik

A 38-year-old female patient living in Wardha contacted the Department of Conservative Dentistry and Endodontics of Sharad Pawar Dental College and Hospital complaining of pain in the right lower back of the jaw in the past month. When the chief complaint was specified, the pain was spontaneous and persisted for minutes after removing the stimulus (usually heat, less often cold). Spontaneous episodes occur randomly throughout the day or night, disrupting sleep and daily activities. The pain was sharp, severe, and difficult for the patient to localize to a specific tooth. The past medical history as well as the past dental history of the patient was nonsignificant. Radiolucency of the enamel, dentin, and pulp, as well as widening of the periodontal ligament space with the right lower tooth, was detected on the intraoral periapical radiograph using the bisecting angle technique. Pulp neurosensibility tests such as electric pulp test and thermal test were performed. After performing an electric pulp test, a delayed response with 46 was seen, and after performing a hot gutta percha test, 46 responded with pain which lingered on the removal of the stimulus. A diagnosis of “symptomatic irreversible pulpitis with apical periodontitis” was made for the right lower first molar (Figure 1).

**Figure 2:**
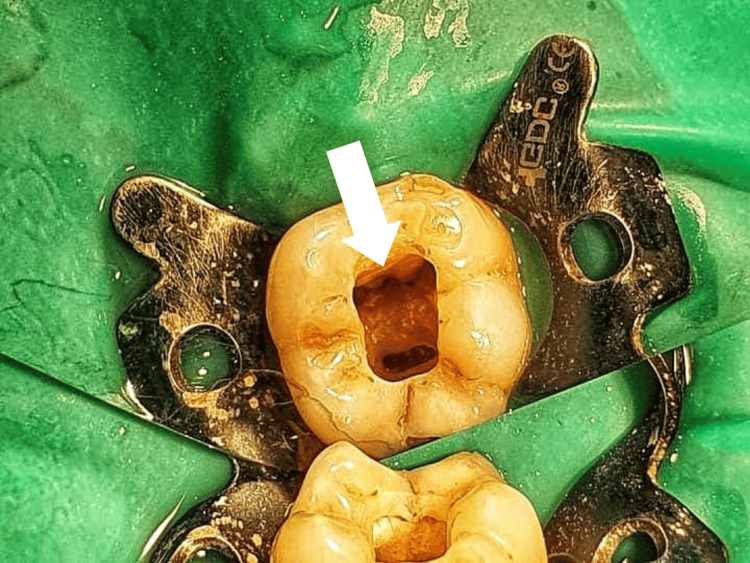
Access opening

The patient received 2% Xylocaine with 1:80,000 adrenaline. Rubber dam isolation was done. A 3.5x loupes were used for magnification and illumination. Round BR-45 (Mani, Japan) and Safe end bur EX-24 (Mani, Japan) were used to prepare the access cavity. After removing the pulp tissue from the chamber, three openings were discovered: mesiobuccal, mesiolingual, and a single wide distal opening in typical places. Using a combination of tactile exploration and enhanced visualization tools like 3.5x dental loupes, pulp chamber floor was probed especially between the mesiobuccal and mesiolingual openings, and a midmesial opening was found (Figure 2).

**Figure 3:**
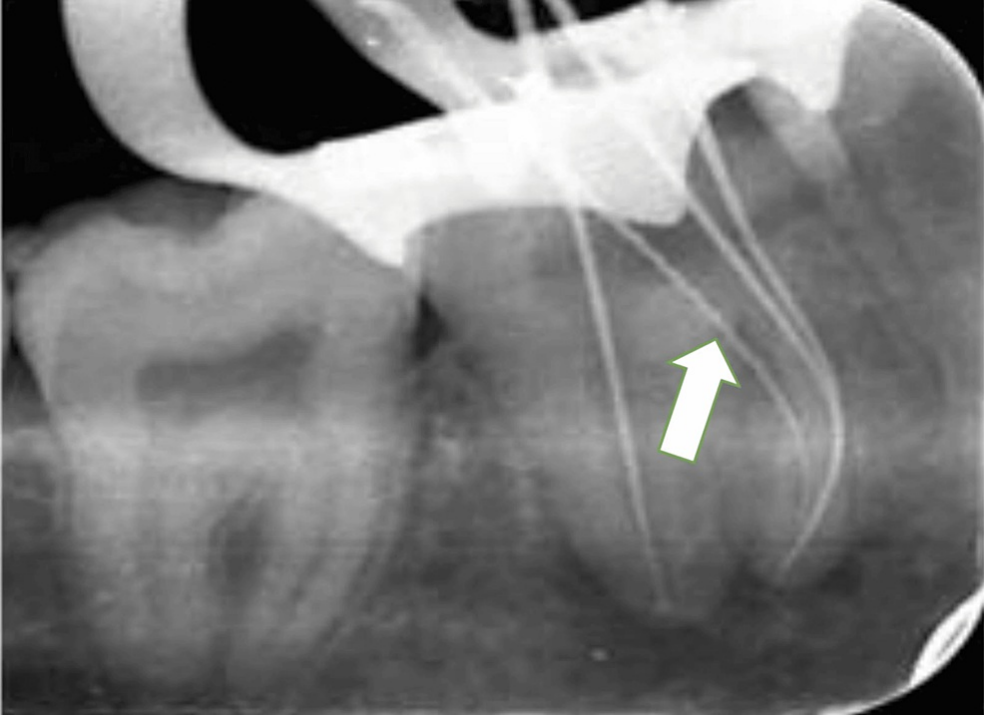
Working length radiograph Image by Dr. Khyati Manik

A #10 K-file (Dentsply Maillefer, Ballaigues, Switzerland) was used to locate all the openings of the root canal. A #15 K-files (Dentsply Maillefer, Ballaigues, Switzerland) were used to measure the working length of all the canals, and then the apex locator (Root ZX, J. Morita Inc) was used to confirm it. The working length was verified by taking an angled radiograph (mesiobuccal 19 mm, mesiolingual 19.5 mm, midmesial 18.5 mm, and a single wide distal canal 19.5 mm) (Figure 3). A Woodpecker Glider (W3, 12/variable taper) was used for preparing the glide path at 350 rpm speed and 1.5 Ncm torque. After this, the orifice was enlarged with W0 (17/.12) (Woodpecker Endo plus Rotary file) at 300 rpm speed and 3 Ncm torque for all the identified canals. The mesiobuccal, mesiolingual, and single wide distal canal were prepared with a NiTi Rotary file (Woodpecker Endo Plus Rotary File) till W4 (25/.06) at 350 rpm speed at 2 N/cm^-2^ torque, whereas MMC was prepared up to W2 (20/.04) at 350 rpm speed and 1.5 N torque. Throughout instrumentation, the canal was filled with a 3% sodium hypochlorite solution as the working solution, which underwent passive ultrasonic activation per canal with EndoX (Waldent Innovations Pvt. Ltd, New Delhi India). In between, a thorough irrigation was performed using normal saline, followed by a final irrigation using 2% chlorhexidine (Dentochlor, Ammdent, India), also activated using the same technique. Calcium hydroxide was placed as an intracanal medicament, and a closed dressing was given with 46.

**Figure 4:**
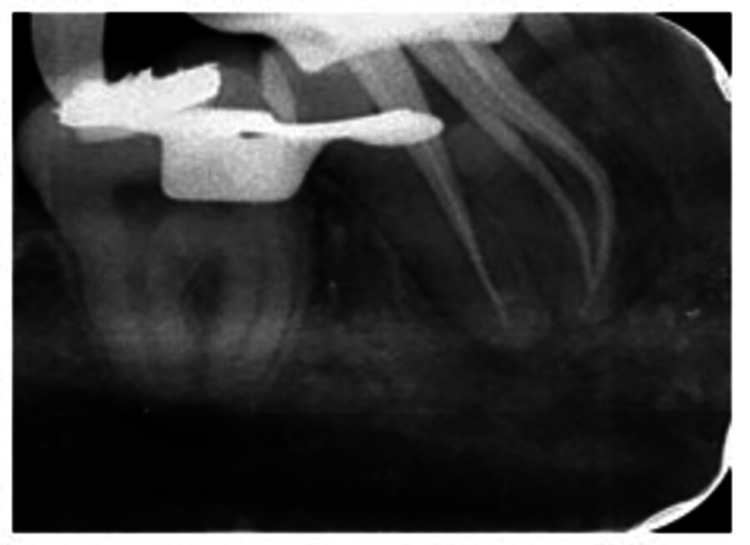
Master cone fit check

On the second appointment, the patient was completely devoid of symptoms. Temporary dressing was removed, and the smear layer was removed with two cycles of 60 s with 17% ethylenediaminetetraacetic acid (EDTA) and 3% sodium hypochlorite (Parcan N, Septodont, UK) with alternate 2 ml NS wash and 2% CHX 3 min passive ultrasonic activation per canal with EndoX (Waldent Innovations Pvt. Ltd, New Delhi India). Moreover, 0.06 # 25 Gutta-percha master cones (Dentsply Sirona) were selected (Figure 4).

**Figure 5:**
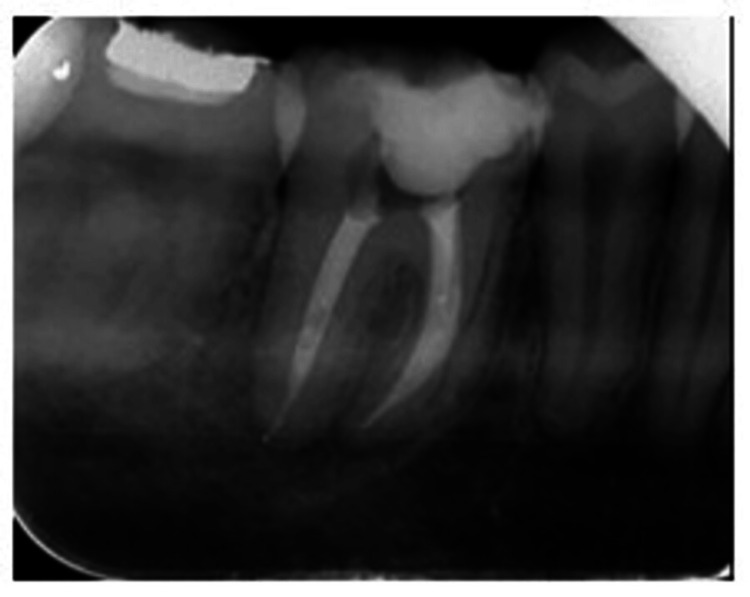
Obturation radiograph

Obturation was carried out with master cones and epoxy resin-based sealer (Dia-ProSeal, Diadent, Canada) (Figure 5).

**Figure 6:**
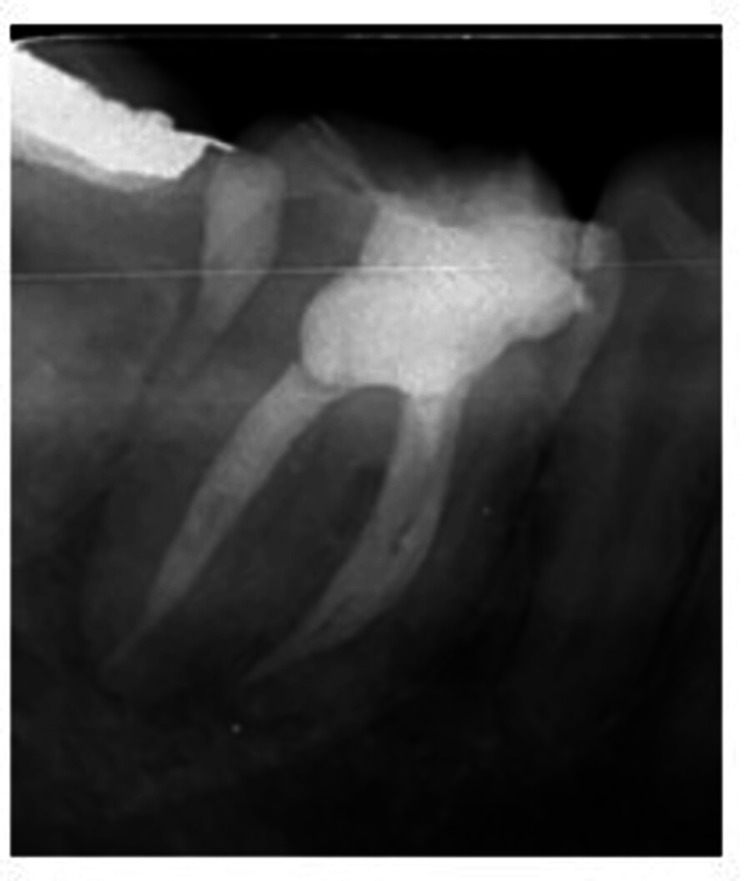
Post-endodontic restoration

Post-endodontic composite restoration (Spectrum, Dentsply, USA) was done (Figure 6).

## Discussion

An MMC in the mandibular first molars is an intriguing aspect of root canal anatomy that has garnered significant attention in endodontics. This additional canal, located between the mesiobuccal and the mesiolingual canal, poses challenges and opportunities in root canal treatment. In this case report, an independent variant of the MMC was found after taking the working length radiograph.

The Indian population showed a prevalence rate of 29.7% for the mandibular first molar and 16% for the mandibular second molar [6]. Goel et al. documented the presence of mandibular first molars that showed three mesial canals in 13.3%, four mesial canals in 3.3%, and three distal canals in only 1.7% of the population [7]. Prevalence tends to be higher in the mandibular first molars compared to the second molars [8,9].

Research studies have suggested that the MMC often exhibits a reduced length compared to the main mesiobuccal and mesiolingual canals, primarily due to its location in the isthmus area between the mesial roots. For example, a study by Pomeranz et al. found that the mean length of the MMC was significantly shorter than that of the main mesiobuccal and mesiolingual canals [4]. This anatomical variation indicates that a shorter working length may be appropriate for the MMC to avoid over-instrumentation and minimize the risk of procedural errors, such as ledge formation or perforation. Moreover, the isthmus region where the MMC is located often presents challenges in instrumentation and disinfection due to its complex morphology and limited accessibility. Studies have demonstrated that isthmuses are more prevalent in the middle and apical thirds of the root, suggesting that focusing instrumentation and obturation efforts in these regions may be more effective in achieving thorough cleaning and sealing of the MMC [10].

The dental surgical microscope and other advancements in technology enhance the visibility of the operative field and make it easier to visualize the canal openings [11]. De Carvalho and Zuolo emphasized the significance of using microscopes to precisely locate root canal openings, which can greatly enhance treatment outcomes [12]. Once identified, effective management of MMCs involves employing appropriate instrumentation techniques to negotiate the narrow and often curved canals. Techniques such as modified access cavity preparation, ultrasonic instrumentation, and micro-guided endodontics can facilitate improved access and negotiation of MMCs [13]. Moreover, using nickel-titanium rotary files with varying tapers and tip sizes can aid in shaping the MMCs while minimizing procedural errors [14]. Throughout the treatment process, continuous irrigation with antimicrobial solutions and chelating agents can help ensure thorough disinfection and removal of pulp tissue from MMCs [15].

In 1925, Hess postulated that mandibular molar roots diverge into one canal and narrow in the middle to form two canals [16]. Root canals are thought to be formed by the vertical dentinal septum, i.e., created by secondary dentine deposition into the canal cavity. MMCs and small extra channels are heavily influenced by age. It was observed that the mesial roots of the mandibular first and second molars mostly had one large canal until the age of 11 and 15, and due to secondary dentin deposition at 30-40 years of age, the canal systems in the apical and middle third of the root was completely established [17]. The importance of diagnostic procedures in identifying additional channels cannot be overemphasized. All methods should be used to identify the mesial canal, such as several preoperative radiographs at various angles, detailed inspection of the pulp chamber floor, grooving through ultrasound tips, staining, visualization of bleeding points, and magnification. Advanced technology like “dental operating microscope” or “cone-beam computed tomography” may also be used [18]. Despite the challenges, successful management of MMCs is achievable with careful treatment planning and execution. Studies have shown that thorough cleaning, shaping, and obturation of MMCs can lead to favorable treatment outcomes and long-term tooth survival.

## Conclusions

Diagnosing and managing MMCs poses a significant challenge in endodontic practice, requiring careful consideration and adept skills. Employing a strategic approach is essential to effectively navigate this intricate aspect of root canal treatment. Once diagnosed, proper management of MMCs demands precision and expertise. Utilizing magnification and illumination tools facilitates enhanced visibility and access to these intricate canal systems. Employing ultrasonic instruments and specialized rotary files designed for negotiating narrow and curved canals can aid in thoroughly cleaning and shaping the MMC. Employing appropriate irrigation protocols and medicaments tailored to disinfect and seal these intricate canal systems is paramount for ensuring successful outcomes. Furthermore, a comprehensive understanding of the anatomical variations and possible aberrations associated with MMCs is crucial. By adopting these strategies, endodontists can confidently navigate the intricacies of MMCs, ultimately enhancing the success rates of root canal treatments and improving patient outcomes. This knowledge enables endodontists to anticipate challenges and adapt their treatment approach accordingly.
